# Use of Organic Materials to Limit the Potential Negative Effect of Nitrogen on Maize in Different Soils

**DOI:** 10.3390/ma15165755

**Published:** 2022-08-20

**Authors:** Marzena S. Brodowska, Mirosław Wyszkowski, Natalia Kordala

**Affiliations:** 1 Department of Agricultural and Environmental Chemistry, University of Life Sciences in Lublin, Akademicka 15 Str., 20-950 Lublin, Poland; 2 Department of Agricultural and Environmental Chemistry, University of Warmia and Mazury in Olsztyn, Łódzki 4 Sq., 10-727 Olsztyn, Poland

**Keywords:** organic materials, nitrogen, soils, maize

## Abstract

This study was launched to test organic materials in the form of humic acids (HA) applied to soil to improve the effect of nitrogen on maize, and to determine an optimal dose of HA, which will be ecologically safe and will counteract potential negative (phytotoxic) influences of excessive nitrogen fertiliser doses, on two soils with different textural composition. The maize plants grown on the loamy sand were characterised by a higher value of the SPAD leaf greenness index, yields, and a lower content of total-N and sulphate sulphur in maize. Urea, and especially UAN, promoted higher SPAD leaf greenness index values during the stem elongation stage and particularly during the tassel emergence stage. The effect of urea on maize yields was positive on both soils, but UAN had a positive effect on this parameter only on the loamy sand. HA tended to increase the SPAD leaf greenness index. The impact of HA on plant height and yields (especially medium dose) was generally positive. However, a negative effect of the interaction of HA with UAN on the plant height and maize yield on the sand was observed. HA caused an increase in the total-N content, and their highest dose also decreased the sulphate sulphur content in maize. The application of HA to soil has a positive influence on the growth and development of plants and can create positive effects by mitigating adverse consequences of intensive agricultural production in the natural environment.

## 1. Introduction

The most important role in maintaining soil fertility is played by organic matter, which is comprised of soil-dwelling organisms as well as products of the decomposition of plant, animal, and microbial residues [1]. Organic matter, both natural and added to soil with fertilisers, and products of organic matter transformations carried out by microorganisms, have a beneficial influence on the physicochemical and biological properties of soil, thereby shaping its production potential [2]. Organic matter is an important factor in counteracting or mitigating the impact of contaminants on plants and soil properties.

The major component of organic matter is humic substances, which are a complex and relatively biodegradation-resistant mixture of brown amorphous colloidal compounds, which arise from the modification (humification) of primary plant tissues or the synthesis of bonds with the participation of microorganisms [3]. According to Stevenson [4], humic substances are a group of heterogenous acids (humic and fulvic ones) and their salts (humates). Humic acids (HA) are built of mainly carbon, hydrogen, and oxygen, in addition to small amounts of nitrogen and sulphur [5]. An important role in humic acids is played by functional groups, which determine the properties of humic acids, such as hydrophilicity, acidity, or ion exchange capacity [5,6,7].

Maize (*Zea mays* L.) is the third most important cereal crop grown worldwide, after wheat and rice. It is a versatile crop as it is grown as fodder, food, and industrial plant [8]. Maize cultivation depletes soil resources of nutrients and requires the application of larger quantities of fertiliser nitrogen than other cereals [9]. However, excessive nitrogen fertilisation ends in a low degree of nitrogen utilisation, thereby causing environmental problems because nitrate ions are leached deep into the soil profile [10]. Hence, it is essential to search for new cultivation technologies, including fertilisation and application of additives, which would have a positive effect on the effectiveness of fertiliser, which would result in increased efficiency and reduced environmental threats [11]. Biostimulants in modern plant cultivation technology are now not only a need but often a necessity, because, apart from fertilisation and protection of plants, they have a positive effect on the yield and quality of crops [12]. The mentioned humic substances, which have a direct and indirect effect on the growth of plants, are counted as biostimulants [13,14] and fit perfectly well in the current trends in agricultural production, expected to be more ecological and ecofriendly.

The indirect impact arises from the amendment of soil properties. Humic substances increase the soil’s water and heat holding capacity, improve its structure, enhance the microbiological activity of the soil, and thereby induce more intensive uptake of macro- and micronutrients by plants [15,16]. A relationship between the content of humic substances in soil and crop yields has been successfully demonstrated [17,18,19]. Humic acids, by forming complexes with cations present in the soil, improve the phytoavailability of phosphorus, magnesium, iron, and zinc [3,20].

The direct effect consists of the induction of biochemical reactions in plant tissues. Humic substances cause an increase in the content of mRNA in cells as well as carrier and structural proteins in leaves, also affecting the hormonal balance of plants (an effect similar to the one produced by auxins) [21], inducing the synthesis of compounds that help plants combat biotic and abiotic stresses [22,23]. Humic substances also have a beneficial influence on the germination of seeds and development of seedlings [24], the growth of roots (especially lateral ones and root hairs) [25,26], and the chlorophyll content of the plant tissues [27]. By increasing the release of organic acids from roots, humic substances promote interactions between plants and useful microorganisms, such as PGPB (plant growth-promoting bacteria) [21,28].

Furthermore, humic acids participate in the biological cycle of elements (carbon and nitrogen) [29], ion exchange, sorption processes, and soil detoxication processes, in addition to stimulating the growth and multiplication of endophytic microorganisms [13]. Other significant roles played by humic substances include their participation in carbon sequestration and greenhouse gas emissions [30], prevention of droughts, and the shaping of soil fertility [31].

Mineral fertilisers are among the most important factors stimulating yields in plant production. However, when applied in excessive quantities, they may contribute to the depletion of organic matter in the soil, decreases in the quality of groundwater and surface waters, and eutrophication of water bodies [1]. The application of mineral fertilisers can also result in a higher content of toxic elements in soil [32,33] and plants [34]. The application of humic substances can be a solution for attaining more efficient use of fertilisers and lesser environmental pollution [22]. Humic acids in combination with nitrogen fertilisers can improve the growth, development, and productivity of crops [20]. Humic acid (HA) enhances the efficiency of fertilisers and prolongs their active impact, reduces nitrogen losses, and accelerates nitrogen uptake and utilisation by plants [35]. Moreover, HA accelerates the rate of organic nitrogen mineralisation in soil, thereby raising the soil resources of available nitrogen [36].

The beneficial effect of the application of organic materials, e.g., humic acids, on soil properties and the growth and development of plants indicate that they can be an effective supporter of typical soil-used fertilisers. However, their impact varied both depending on the species of the plant, the soil type, and the fertiliser type. This is especially important in the case of nitrogen fertilisers that affect plants quickly and very effectively, but at the same time can pose a serious threat to the environment.

Because of the high consumption of nitrogen fertilisers, it is extremely important to apply nitrogen fertilisers rationally, in a way that will not pose a threat to the natural environment. Hence, this study was launched to test organic materials in the form of humic acids applied to soil to improve the effect of nitrogen on maize, and to determine an optimal dose of HA, which will be ecologically safe and will counteract potential negative (phytotoxic) influences of excessive nitrogen fertiliser doses. The model study was carried out on two soils with different textural compositions.

## 2. Materials and Methods

### 2.1. Methodology of the Plant Growing Experiment

**Figure 1:**
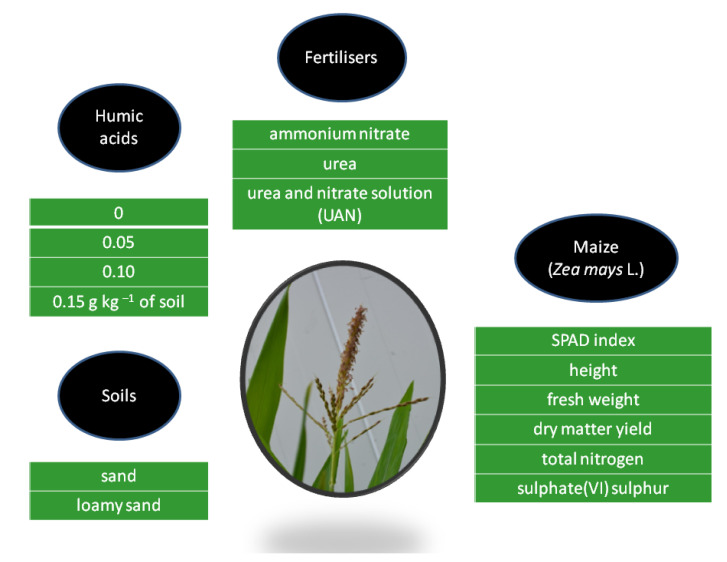
Experiment scheme.

The study was based on a plant-growing pot experiment. It was carried out in a three-factorial experiment design. The experimental factors were the application of organic material in the form of humic acids, different types of nitrogen fertilisers, and 2 soils with different textural compositions (Figure 1).

Humic acids were applied in the following doses: 0, 0.05, 0.10 and 0.15 g kg^−1^ of soil in order to enhance the influence of nitrogen fertilisation on plants. HA was applied three times, in identical doses, during the maize growing period: before sowing, in the fifth leaf stage, and in the stem intensive growth stage. The experiment was conducted in three series: with ammonium nitrate (34% N), urea (46% N), and urea and nitrate solution UAN (32% N). Identical doses of nitrogen were applied to all the pots, 160 mg N kg^−1^ of soil. The experiment was set up on two types of soil: sand—soil I (>0.05 mm sand—91.88%, 0.002–0.05 mm silt—7.44% and <0.002 mm clay—0.68%; total-N content—5.79 g kg^−1^ of soil), and loamy sand—soil II (>0.05 mm sand—77.55%, 0.002–0.05 mm silt—19.95% and <0.002 mm clay—2.50%; total-N content—6.20 g kg^−1^ of soil) [37]. The soils (sand and loamy sand) were taken from surface layers 0–25 cm of soil. Before placing it in pots, the soil was sieved through a sieve with a diameter of 1 cm. Before sowing maize in each research series 60 mg P and 170 mg K per kg of soil in the form of Super FosDar 40 and KCl were used. Humic acids were applied as an organic-mineral fertiliser called Humik. Humik contains: humic acids 25%, organic carbon 22%, amino acids 10%, betaine 10%, nitrogen (N) 4%, phosphorus (P_2_O_5_) 0.10%, potassium oxide (K_2_O) 5%, magnesium (MgO)—0.5%, vitamin B1 3 mg kg^−1^, vitamin B2 95 mg kg^−1^. The content of organic matter was 52%. Humik is a liquid with dark amber colour, a relative density of 1.30–1.35 g cm^3^, and pH 5–6.

Nitrogen, phosphorus, and potassium fertilisers as well as the first dose of humic acids were carefully mixed with soil when the experiment was started, and 9 kg batches of soil were put into pots. Next, the maize cultivar Kadryl was sown. The density of plants was 6 plants per pot. During the vegetative growth of maize, the leaf greenness index SPAD was measured three times, the stages of the fifth leaf unfolded, intensive stem elongation and tasselling. The soil moisture was maintained at the same level of 60% maximum water holding capacity throughout the experiment. The soil moisture was controlled by weighing pots in which plants were grown. Maize was harvested at the end of tassel emergence (BBCH 59). The plant height and yield of aerial parts of maize were determined on harvest, and next plant samples were collected for laboratory analyses.

### 2.2. Methodology of Laboratory and Statistical Analyses

Measurements of the SPAD leaf greenness index during the growth of plants were made with a chlorophyll meter Minolta SPAD-502Plus (Konica Minolta Sensing Europe B.V., Nieuwegein, The Netherlands). The plant samples collected during the maize harvest were cut and dried at a temperature of 60 °C and ground. The total nitrogen content was determined with Kjeldahl’s method [38]. Plant samples were wet mineralised in concentrated sulphuric acid (H_2_SO_4_ p.a. purity grade) in a Speed-Digester K-439 (BÜCHI Labortechnik AG, Flawil, Switzerland) [39]. Next, the samples were distilled in a KjelFlex K-355 Kjeldahl distiller (Büchi Labortechnik AG, Flawil, Szwajcaria) and titrated using a TitroLine 7000 (Xylem An-Cylytics, Weilheim, Germany). The content of sulphate (VI) sulphur was determined with the nephelometric method after extracting the plant material with 2% CH_3_COOH with added active carbon [40]. The soil texture (granulometric) composition was determined with the laser method using a Mastersizer 3000 (Malvern Instruments Ltd., Worcestershire, UK) [41].

The results were processed statistically in Statistica [42] according to the three-factorial analysis of variance ANOVA with the HSD Tukey’s test (*p* ≤ 0.01), PCA and percentage of variance observed with the ANOVA method.

## 3. Results

**Figure 2:**
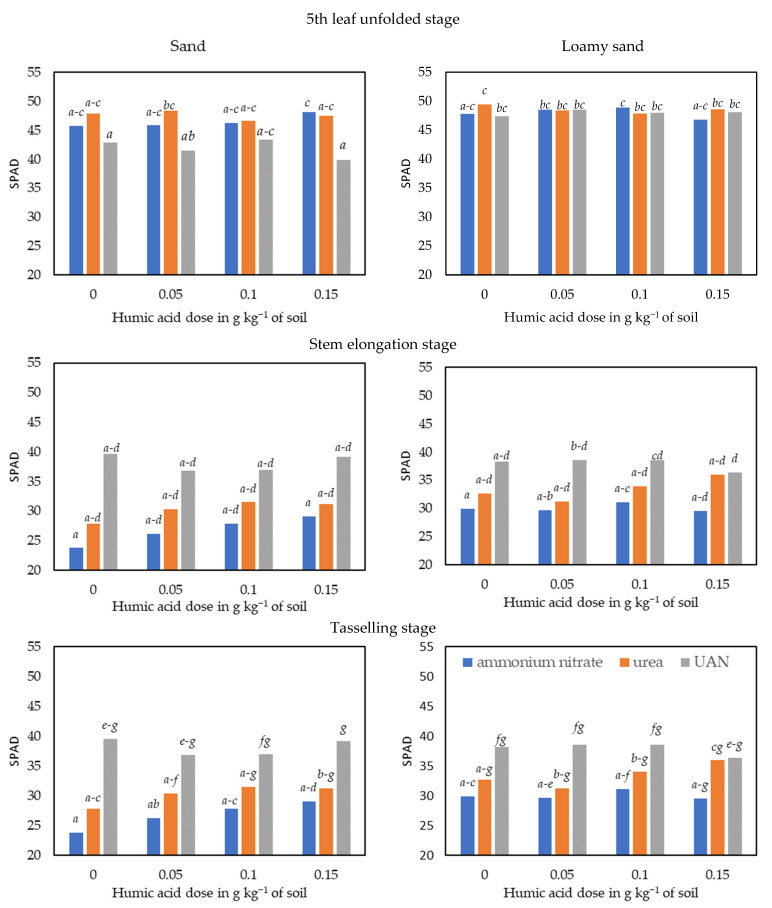
SPAD index in maize—*Zea mays* L. vegetation stages of the 5th leaf unfolded, stem elongation, and tasselling. Values with different letters (*a–g*) are significantly different at *p* ≤ 0.01 (Anova, Tukey’s HSD test).

**Figure 3:**
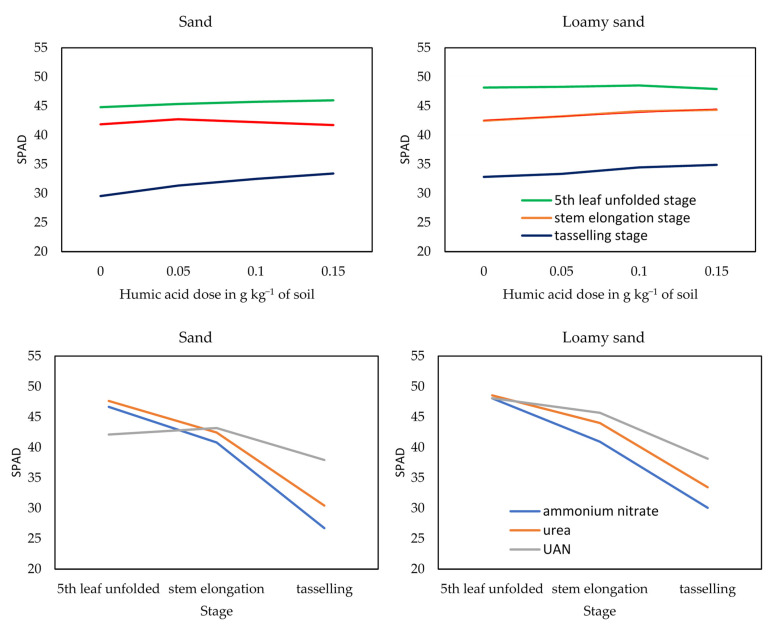
Effect of humic acid and fertiliser form on SPAD index in maize—*Zea mays* L. (averages from series).

The factors analysed in the experiment (type of soil and form of nitrogen fertilisers, organic materials in the form of humic acids applied to enhance the effectiveness of mineral fertilisers) affected the SPAD leaf greenness index, the height and yields of maize (*Zea mays* L.) and the content of total nitrogen and sulphate sulphur in the aerial parts of this crop (Figure 2 and Figure 3, Table 1 and Table 2).

The SPAD leaf greenness index values determined for the maize decreased in the course of the vegetative growth of the plants, especially in the series with ammonium nitrate and with urea, more on the sand than on the loamy sand (Figure 2 and Figure 3). The SPAD index of maize was higher on the loamy sand than on the sand, on average by 3% (stem elongation stage) to 7% (tassel emergence stage). In the fifth leaf stage and the maize stem elongation stage, differences between the two types of soil were greater in the series with water solution of urea and ammonium nitrate (UAN), 14% and 6%, respectively, while being bigger during the tassel emergence stage in the objects with urea—10%, and with ammonium nitrate—13%. Urea and particularly UAN promoted an increase in the SPAD values for maize during the stem elongation stage and, especially, the tassel emergence stage. In the tassel emergence stage, the SPAD values for maize in the series with urea and with UAN were higher by 13% and 43% on the sand, and by 11% and 26% on the loamy sand than in the series fertilised with ammonium nitrate.

The influence of humic acids on the SPAD leaf greenness index determined for maize also depended on the plant’s vegetative growth stage (Figure 2 and Figure 3). The smallest changes were noted in the fifth unfolded leaf stage, particularly on the loamy sand, while the biggest ones appeared during the stage of tassel emergence on the sand. In the maize fifth unfolded growth stage, the application of humic acids to soil contributed to an increase in the SPAD index by 7% in the series with ammonium nitrate and by 5% (second dose) in the objects with UAN. During the stem elongation stage, analogous effects of HA were only observed under the influence of UAN and urea on the loamy sand. These substances resulted in an increase in the maize’s SPAD values by 5% and 6%, respectively, compared with the control objects (without HA). The most beneficial effect of HA on the SPAD leaf greenness index of maize was demonstrated in the tassel emergence stage, with the strongest effect on the sand produced by ammonium nitrate (+22%) and on the loamy sand—by urea (+13%). Humic acids produced analogous albeit weaker effects on the SPAD values determined for maize in the series with UAN (+6%) and urea (+15%) on the sand, and the series with ammonium nitrate (+8%) on the loamy sand.

The influence of the type of soil on the height of maize plants was relatively weak, and the plant height was greater by 11% on the loamy sand than on the sand only in the series with UAN (Table 1). On the sand, urea contributed to a small, 4% increase in plant height, while UAN resulted in the lowering of this trait by 9% in comparison with the series treated with ammonium nitrate.

The impact of HA on the height of maize plants was most positive in the series with ammonium nitrate (Table 1). There, HA led to a 7% increase in the plant height on the sand, relative to the control (without HA). An analogous effect was noted on the loamy sand in objects with ammonium nitrate and UAN, but only under the influence of the first and second dose of humic acids. However, it is worth mentioning that humic acids had a distinctly negative effect on the plant height in objects with UAN on the sand, which was not indifferent to yields of maize aerial parts.

The yield of aerial organs of maize proved to depend on the type of soil and was higher by an average of 12% in terms of fresh mass and 15% for dry matter on the loamy sand than on the sand (Table 1). Differences in yields between the series fertilised with different nitrogen fertilisers were demonstrated on both soils. Urea had a more positive effect than ammonium nitrate on maize yields obtained from both soils, while UAN affected maize yields positively only on the loamy sand. An increase in the fresh and dry matter yields of maize in response to urea was on average 3% and 11%, respectively, on the sand versus 7% and 6% on the loamy sand and compared with a 12% and 12% increase, respectively, on the loamy sand after UAN had been applied. The yield of maize fresh matter yield in the UAN fertilised objects on the sand was on average 16% (fresh mass) and 25% (dry mass) lower than in the objects with ammonium nitrate.

In most cases, the application of humic acids had a positive effect on maize yields (Table 1). In most series, the optimal dose of HA proved to be the medium one, especially in terms of the dry matter yields. It was only in the series with urea on the sand that the first dose of HA had the best effect, while on the loamy sand, the highest yields were obtained following the medium dose of humic acids. These doses resulted in dry matter yields of maize being higher by 21% and 14%, respectively, relative to the control (without humic acids). The impact of humic acids on the fresh matter yield of maize in the series with urea and on the fresh and dry matter yield in the series with ammonium nitrate on both soils was weaker although positive as well. The influence of HA on the yields of the fresh and dry mass of maize aerial parts in the series fertilised with UAN on the sand was distinctly negative while being positive on the loamy sand, although only when humic acids had been applied in the medium dose.

The average content of total nitrogen and sulphate sulphur in the aerial parts of the maize grown on the loamy sand was 10% and 6% lower on average than in the maize cultivated on the sand (Table 2). Both the total nitrogen and sulphate sulphur concentrations in maize aerial organs were differentiated by the form of applied nitrogen fertilisers. In comparison with the series fertilised with ammonium nitrate, the total nitrogen content in maize was higher in the objects with UAN by 78% (sand) and by 15% (loamy sand), and in the series with urea—by 16% (sand). Reverse relationships were demonstrated in maize grown on the loamy sand fertilised with urea. Urea also resulted in a decrease in the sulphate sulphur content in maize, by about 10% on average in both soils, while UAN was responsible for a 22% decline in pots filled with the loamy sand, in relation to objects with ammonium nitrate. The effect of UAN on the content of sulphate sulphur in the aerial parts of maize grown on the sand was weak.

Humic acids, and most often their medium (objects with ammonium nitrate and urea on the sand, and with urea and UAN on the loamy sand) or highest dose (objects with UAN on the sand and with ammonium nitrate on the loamy sand), led to an increase in the total nitrogen content of maize aerial organs (Table 2). An increase in the total nitrogen content induced by the presence of humic acids most often fell in the range of 19% to 23%, except for the series with urea (+7%) and UAN (+80%) on the sand. The highest dose of HA contributed to the reduction in the sulphate sulphur content in the maize aerial parts in most experimental series, with an analogous effect achieved in the objects with urea even by the lowest HA dose. An exception was the series with UAN on the sand, where reverse relationships were observed. The negative effect of humic acids on the content of sulphate sulphur in the maize aerial parts in the objects fertilised with urea was significantly stronger than in the other series of the experiment.

**Figure 4:**
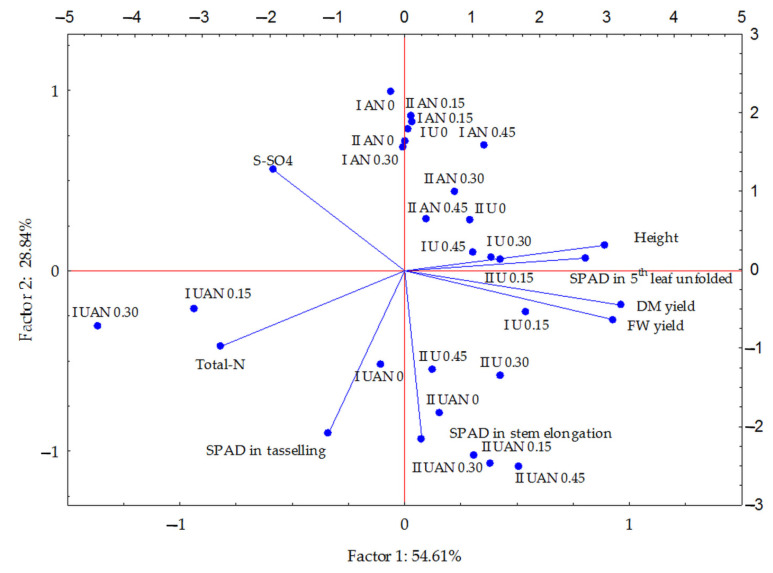
Yield, biometric features, and content of total-N and S-SO_4_ in the aerial parts of maize (*Zea mays* L.) calculated with the PCA method. Key: vectors represent variables (SPAD in stages of the 5th leaf unfolded, stem elongation, and tasselling; height; aerial parts fresh weight and dry matter yield; content of total-N and S-SO_4_); points show the samples with elements (I—sand, II—loamy sand; AN—ammonium nitrate, U—urea, UAN—solution of urea and ammonium nitrate; 0, 0.05, 0.10, and 0.15 g humic acid per pot).

The PCA results (Figure 4) indicate that the parameters concerning the SPAD leaf greenness index values during the tassel emergence stage and stem elongation stage, and the content of sulphate sulphur in the aerial parts of maize were comprised in the first group, representing 54.61%, while the plant height, SPAD values in the fifth leaf unfolded stage, fresh and dry matter yield of the aerial parts of maize and total nitrogen content composed the second group, making up 28.84% of the correlations of the data set.

Most vectors (except the SPAD in the fifth leaf unfolded stage and the content of S-SO_4_) had similar lengths, i.e., a similar impact on the analysed factors. The strongest positive correlations appeared between the yields of fresh and dry matter of aerial parts, the height of the plants versus the SPAD leaf greenness index in the fifth unfolded leaf stage, while weaker ones were between the SPAD index in the tassel emergence stage versus the SPAD index in the maize stem elongation stage. Relatively weak negative correlations were detected between the total nitrogen content versus plant height, and the SPAD index value in the fifth unfolded leaf stage, as well as between the total nitrogen and sulphate sulphur content versus the fresh and dry matter content of the aerial parts of maize. The scattering of data in Figure 4 confirms a stronger effect of humic acids (especially in medium and highest doses) in the objects with urea and UAN than in the series with ammonium nitrate on the analysed parameters.

**Figure 5:**
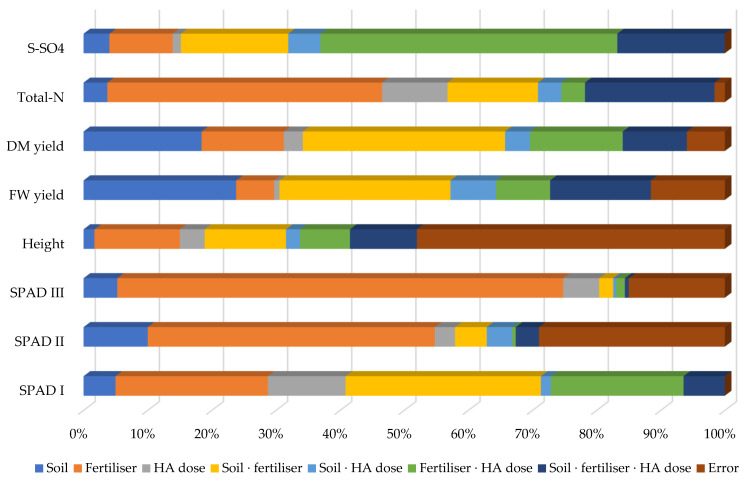
Relatively effect of factors on yield and other parameters of maize—*Zea mays* L. (in per cent). Key: SPAD I—SPAD in 5th leaf unfolded; SPAD II—SPAD in stem elongation; SPAD III—SPAD in tasselling; FW—fresh weight; DM—dry matter; HA—humic acid.

The percentage of observed variance, illustrated in Figure 5, is a statistical presentation of the cumulative effect of the analysed factors on maize.

The form of nitrogen supplied as fertiliser had the strongest effect on the SPAD leaf greenness index in the tassel emergence stage (69.5%) and stem elongation stage (44.8%), total nitrogen content (42.9%), the SPAD index in the fifth unfolded leaf stage (23.8%), plant height (13.3%) and sulphate sulphur content (9.9%). The type of soil had the strongest effect on the yield of fresh mass (23.8%) and dry mass (18.4%) of maize aerial parts. The influence of humic acids was weaker than that of nitrogen fertilisation and type of soil, but their effect on the SPAD leaf greenness index in the fifth unfolded leaf stage (12.1%) and total nitrogen content in the maize aerial parts (10.2%) is noteworthy. Humic acids had the strongest effect when acting in combination with nitrogen fertilisers, and the impact of the type of soil was also the strongest in interaction with these fertilisers.

## 4. Discussion

Due to the rapid growth of the human population in the world, an infinite enlargement of maize plantations is impossible. Hence, the only way to raise the volume of maize yields is by enhancing the yield potential of the existing maize genotypes through the development of various integrated practices for the management of nutrients [43].

Nitrogen is one of the basic yield-promoting elements [44]. The nitrogen nutrition status of plants is of key importance in the growth, development, photosynthetic activity, and productivity of crops, such as maize [45]. In order to obtain high yields of maize, mineral fertilisers, especially nitrogen ones, are added to the soil. However, their application has certain drawbacks, for example, the low degree of nitrogen utilisation [46] or susceptibility to instant dissolution and rapid dispersion [47], which may pose a threat of excessive pollution of groundwater and surface water bodies. The value of recovery (utilisation) of nitrogen from urea in soil by the plant systems rarely exceeds 50% of the applied nitrogen [48]. The low nitrogen utilisation efficiency (NUE) is mostly due to the processes of leaching, denitrification, and volatilisation of nitrogen in the form of oxides (NO_x_) [49].

The effective management of nitrogen supply to agricultural crops (minimising losses of nitrogen from the soil and maximising its assimilation by plants) can bring about measurable economic benefits to producers. It is estimated that increasing the NUE indicator by as little as 1% may lead to savings of up to 1.1 billion USD annually from fertiliser consumption [50,51]. This is the reason why it is important to develop new methods for the improvement of the efficiency of nitrogen utilisation in crop plantations, so as to ensure high yields while lowering the requirements concerning doses of applied nitrogen fertilisers [51]. The combined application of inorganic fertiliser with organic additives is an effective strategy for ensuring sustainable agricultural production in the context of production, economy, and the natural environment [52].

In this study, the application of humic acids had a positive effect on the maize yield quality parameters selected for analysis. The combined application of humic acids and UAN on the loamy sand proved to be most effective in terms of the volume of maize yields and plant height. It was also observed that the application of urea and UAN had a more beneficial influence on the mentioned maize traits than ammonium nitrate. Similar conclusions were drawn by Azeem et al. [53]. In their experiment, the combined application of nitrogen (N) (as urea) and humic acid (HA) considerably improved the physiology of maize and maize yield parameters. The best effects were achieved by the cited researchers after the application of humic acid in a dose of 4.5 kg ha^−1^. In this variant, the plant height, biomass of stems and yield of grain were higher by 11.6%, 5.6%, and 14.6%, respectively, relative to the series with the smallest HA dose (1.5 kg ha^−1^). Similar results were also obtained by Azeem et al. [54] and Guo et al. [30]. Baldotto et al. [55] noted a 15% increase in maize grain yield following the application of HA. According to those scholars, humic acid had a positive influence on the development of maize and the formation of grain yield by increasing the content of organic matter and availability of total nitrogen in the soil, which resulted in a higher uptake of nitrogen by the crop. Niaz et al. [56] also demonstrated that the application of HA and N largely improved the utilisation of nitrogen in plants and soil, enhancing the yield of maize grown on calcareous soil. This beneficial effect is probably associated with the formation of a high stability complex, following the application of urea with humic acid, which improves the availability of NH_4_^+^-N and NO_3_^−^-N in soil, as well as raising the efficiency of nitrogen utilisation and reducing nitrogen losses [57,58,59].

Khan et al. [43] evaluated the effect of adding humic acid (0, 0.6, 1.2 and 1.8 kg ha^−1^) and nitrogen fertilisation (0 and 120 kg ha^−1^) on yields produced by two maize cultivars, Jalal and Iqbal. All the applied doses of humic acid added to soil, in combination with nitrogen, had a beneficial effect on the height of plants and the quality of yields of the two maize cultivars. However, the biggest changes were noted in the series with the highest dose of HA (1.8 kg ha^−1^). Relative to the control (no fertilisation; 0 kg HA + 0 kg N ha^−1^), the above treatment improved the 1000 kernels weight (by 15%), yield index (by over 30%), the content of nitrogen in grain (by 20%) and plant height (a difference from 15.5 to 17.8 cm).

In this experiment, the content of nitrogen in the maize aerial biomass was also positively correlated with the addition of humic acids, and this effect was more unequivocal in the series with UAN or with urea. However, a reverse relationship was noted in the case of sulphur content. Humic acids affected positively the greenness of maize leaves during the crop’s vegetative growth. The best results on the sand were achieved when humic acids and ammonium nitrate were applied, and on the loamy sand—following the application of humic acids and urea. When the nitrogen fertilisers were applied without HA, urea or UAN had a stronger influence on the leaf greenness index than ammonium nitrate.

The positive effect of the interaction of humic acids with urea in maize cultivation was also shown by Pei et al. [60]. They demonstrated that it was possible to decrease the conventional nitrogen fertilisation doses by 15% when humic acid in an amount of 3000 kg hm^−2^ was added. This fertilisation treatment turned out to be most effective, improving the agronomic traits of maize, yield index (by 13.8%), and nitrogen utilisation efficiency (by 59.9%). The height and diameter of plants increased by 3.7% and 2.3%, respectively. The researchers also reported an increase in the nitrogen content in grain (by 2.7%), the accumulated amount of nitrogen in grain (by 26.0%) and total nitrogen content in maize aerial biomass (by 10.7%).

The combined application of urea solution (4.0 g pot^−1^) and fulvic acid (5.4 g pot ^−1^) tested by Gao et al. [61] improved the maize yield and NUE values by 16.9% and 24.3%, respectively, in a two-year pot experiment. Under these conditions, the quoted authors also noted a rise in the NH_4_^+^ content in soil by 12.1% and a positive influence of fulvic acid on the soil structure and availability of nutrients. The application of fulvic acid stimulated the activity of enzymes associated with nitrogen metabolism, promoted the efficiency of photosynthesis, and affected the expression of genes encoding endogenous hormones in the plant.

Humic substances stimulate the activity of growth hormones in plants, induce the course of various biochemical processes in cells, and significantly influence the content of photosynthetic pigments in plants [62]. Moreover, they raise the uptake of nitrogen from soil by plants, accelerate their vegetative growth, development of leaves and size of leaves [59]. The improved supply of nitrogen to plants significantly affects the longevity of leaves, which largely determines the biomass gains and yielding of plants [54]. This way, humic substances show a positive, stimulating effect on the quality of crops and crop yields.

The application of humic acid (foliar application, 13 mg dm^−3^) and half of the recommended dose of nitrogen fertiliser in wheat cultivation by El-Bassioung et al. [63] resulted in a considerable increase in quantities of soluble solid substances and total carbohydrates in wheat biomass. According to these researchers, the mentioned increase arose from the growing efficiency of photosynthesis in response to the treatment with humic acid. This conclusion is substantiated by another finding made in that study, namely increased content of chlorophyll a and b, as well as carotenoids in leaves of the analysed plant relative to the control series (without fertilisation). The application of humic acid probably caused a rise in the synthesis of chlorophyll or delayed its degradation in primary and lateral leaves. The positive effect of humic acids on the SPAD leaf greenness index, dictated by the content of chlorophyll in plants, was also demonstrated in the current experiment, particularly in the objects fertilised with UAN.

The positive effect of the combined fertilisation with potassium nitrate (100 mg dm^−3^) and humic acid (40 mg dm^−3^) on the vegetative and reproductive growth, total yield, the content of chlorophyll and nitrogen in leaves in the cultivation of potted cucumbers was also shown by Kazemi [64]. There are also many reports on benefits gained from the application of humic substances in the cultivation of commercial crops, such as sugar cane [3], kiwi [65], hot pepper [66], or strawberry [5].

In the experiment completed by Reeza et al. [67], the addition of humic substances to soil decreased the losses of ammonia by 13% to 25%, relative to the series with urea alone. Furthermore, the application of fulvic acid elevated the content of NH_4_^+^ and NO_3_^−^ as well as K^+^ and Na^+^ ions in the soil. According to these authors, the deceleration of urea hydrolysis could have been caused by a temporary decrease in soil pH, due to the low pH of humic acid (1.89–2.27) and fulvic acid (1.14–1.20), increased content of exchangeable cations in soil with added humic substances and a higher amount of generated NH_4_^+^ than NH_3_^−^ ions.

The effect of humic substances and various nitrogen fertilisers on plants depends on the kind and quality of the soil. In our research, better results were achieved on loamy sand than on sand. It is difficult to select the best nitrogen fertiliser for all kinds of soils because it depends on many factors (soil properties, meteorological conditions, species and plant variety, etc.). Studies of other cited authors were usually performed on one type of soil. Therefore, there is a great difficulty in an unambiguous comparison of their (humic substances, fertilisers) impact on plants on different soil types, due to the impact of other factors.

The application of humic acids as biostimulants in agrotechnology can limit losses of soil organic matter and help to ensure the long-lasting stability of crop yields. The addition of humic acids also makes it possible to reduce doses of mineral fertilisers or to replace synthetic growth regulators. All these effects correspond well with the concept of sustainable agriculture. Therefore, an increase in the use of humic substances and soil amending preparations containing such substances can be expected in the future, which encourages more research in this field.

## 5. Conclusions

The maize plants grown on the loamy sand were characterised by a higher value of the SPAD leaf greenness index, fresh matter yield, dry matter yield, and a lower content of total nitrogen and sulphate sulphur in maize aerial parts.

Urea, and especially UAN, promoted higher SPAD leaf greenness index values during the stem elongation stage and particularly during the tassel emergence stage. The effect of urea on maize aerial part yields was positive on both soils, but UAN had a positive effect on this parameter only on the loamy sand. The effect of these substances on the content of total nitrogen and sulphate sulphur in maize plants depended on the type of soil and experimental series.

Humic acids tended to increase the SPAD leaf greenness index, especially in the tassel emergence stage, and more evidently in the series with ammonium nitrate and urea than in the one with UAN. The impact of HA on plant height and yields was generally positive, and the optimal dose of HA in most series was the medium one. However, a negative effect of the interaction of HA with UAN on the plant height and maize yield on the sand was observed. Humic acids caused an increase in the total nitrogen content, and their highest dose also decreased the sulphate sulphur content in the maize aerial parts.

The application of humic acids to soil has a positive influence on the growth and development of plants and can create positive effects by mitigating adverse consequences of intensive agricultural production in the natural environment.

## Figures and Tables

**Table 1:** Height, fresh weight (FW), and dry matter (DM) yield of aerial parts of maize (*Zea mays* L.).

Humic Acid Dose g kg^−1^ of Soil	Sand				Loamy Sand			
	Ammonium Nitrate	Urea	UAN	Average	Ammonium Nitrate	Urea	UAN	Average
Height (cm)								
0	192.9 *^ab^*	209.7 *^ab^*	204.7 *^ab^*	202.4 *^A^*	195.1 *^ab^*	206.7 *^ab^*	193.7 *^ab^*	198.5 *^A^*
0.05	204.0 *^ab^*	212.1 *^ab^*	184.9 *^ab^*	200.3 *^A^*	202.6 *^ab^*	210.9 *^ab^*	206.0 *^ab^*	206.5 *^A^*
0.10	205.8 *^ab^*	213.4 *^b^*	180.1 *^ab^*	199.8 *^A^*	208.3 *^ab^*	203.3 *^ab^*	209.9 *^ab^*	207.2 *^A^*
0.15	206.1 *^ab^*	207.6 *^ab^*	163.3 *^a^*	192.3 *^A^*	205.8 *^ab^*	191.1 *^ab^*	201.0 *^ab^*	199.3 *^A^*
Average	202.2 *^AB^*	210.7 *^A^*	183.3 *^B^*	198.7 *^A^*	203.0 *^AB^*	203.0 *^A^*	202.7 *^AB^*	202.9 *^A^*
*r*	0.853	−0.251	−0.978	−0.904	0.852	−0.824	0.476	0.086
Aerial parts fresh weight yield (g pot^−1^)								
0	738.9 *^cd^*	753.0 *^de^*	816.5 *^de^*	769.5 *^ACD^*	745.6 *^cd^*	799.0 *^de^*	805.6 *^de^*	783.4 *^AD^*
0.05	741.0 *^cd^*	786.4 *^de^*	641.8 *^bc^*	723.1 *^B^^–D^*	758.7 *^cd^*	810.7 *^de^*	838.6 *^de^*	802.7 *^A^*
0.10	754.6 *^cd^*	783.0 *^de^*	581.1 *^ab^*	706.2 *^BC^*	774.8 *^c^^–e^*	828.9 *^de^*	900.2 *^e^*	834.6 *^A^*
0.15	783.7 *^de^*	782.8 *^de^*	489.4 *^a^*	685.3 *^B^*	762.8 *^c^^–e^*	829.6 *^de^*	876.6 *^de^*	823.0 *^A^*
Average	754.6 *^A^*	776.3 *^AB^*	632.2 *^D^*	721.0 *^A^*	760.5 *^A^*	817.1 *^BC^*	855.3 *^C^*	810.9 *^B^*
*r*	0.926	0.711	−0.975	−0.971	0.726	0.955	0.850	0.861
Aerial parts dry matter yield (g pot^−1^)								
0.05	127.0 *^d^^–f^*	152.7 *^gh^*	100.0 *^bc^*	126.6 *^AC^*	131.8 *^d^^–h^*	140.3 *^d-h^*	148.1 *^gh^*	140.1 *^B^*
0.10	129.5 *^d^^–f^*	141.6 *^d^^–h^*	84.7 *^ab^*	118.6 *^CD^*	134.8 *^d^^–h^*	149.4 *^gh^*	148.9 *^f^^–h^*	144.4 *^A^*
0.15	128.0 *^d^^–f^*	140.7 *^d^^–h^*	62.9 *^a^*	110.5 *^D^*	132.3 *^d^^–h^*	133.6 *^d-h^*	144.6 *^e^^–h^*	136.8 *^AB^*
Average	126.4 *^B^*	140.3 *^AC^*	95.4 *^D^*	120.7 *^A^*	130.9 *^BC^*	138.7 *^A^*	146.8 *^A^*	138.8 *^B^*
*r*	0.814	0.389	−0.987	−0.951	0.769	0.251	−0.100	0.384

*r*—correlation coefficient. Values with different letters (*^a–h^* and *^A–D^*) are significantly different at *p* ≤ 0.01 (Anova, Tukey’s HSD test).

**Table 2:** Content of total-N and sulphate sulphur (VI) in maize—*Zea mays* L. (g kg^−1^ DM).

Humic Acid Dose g kg^−1^ of Soil	Sand				Loamy Sand			
	Ammonium Nitrate	Urea	UAN	Average	Ammonium Nitrate	Urea	UAN	Average
Total-N content (g kg^−1^ DM)								
0	8.59 *^ab^*	10.17 *^b^^–g^*	11.85 *^h^^–j^*	10.20 *^AB^*	9.33 *^a^^–e^*	8.87 *^a^^–c^*	11.57 *^g^^–j^*	9.92 *^A^*
0.05	8.87 *^a-c^*	10.55 *^d^^–h^*	12.97 *^i^^–k^*	10.80 *^B^*	9.89 *^b^^–f^*	9.05 *^a^^–d^*	13.16 *^jk^*	10.70 *^AB^*
0.10	10.55 *^d-h^*	10.92 *^e^^–h^*	18.01 *^l^*	13.16 *^C^*	11.11 *^f^^–h^*	10.64 *^d^^–h^*	14.00 *^k^*	11.92 *^D^*
0.15	8.12 *^a^*	10.36 *^c^^–h^*	21.28 *^m^*	13.25 *^C^*	11.48 *^f-i^*	10.08 *^b^^–g^*	9.33 *^a-e^*	10.30 *^AB^*
Average	9.03 *^A^*	10.50 *^B^*	16.03 *^D^*	11.85 *^B^*	10.45 *^B^*	9.66 *^A^*	12.02 *^C^*	10.71 *^A^*
*r*	0.033	0.379	0.976	0.940	0.980	0.799	−0.370	0.349
Sulphate(VI) sulphur content (g S-SO_4_ kg^−1^ DM)								
0	0.118 *^a^*	0.147 *^a^*	0.099 *^a^*	0.121 *^A^*	0.120 *^a^*	0.128 *^a^*	0.093 *^a^*	0.114 *^A^*
0.05	0.127 *^a^*	0.092 *^a^*	0.122 *^a^*	0.114 *^A^*	0.146 *^a^*	0.111 *^a^*	0.102 *^a^*	0.120 *^A^*
0.10	0.134 *^a^*	0.099 *^a^*	0.131 *^a^*	0.121 *^A^*	0.120 *^a^*	0.098 *^a^*	0.112 *^a^*	0.110 *^A^*
0.15	0.108 *^a^*	0.103 *^a^*	0.148 *^a^*	0.120 *^A^*	0.112 *^a^*	0.116 *^a^*	0.086 *^a^*	0.105 *^A^*
Average	0.122 *^A^*	0.110 *^A^*	0.125 *^A^*	0.119 *^A^*	0.125 *^A^*	0.113 *^A^*	0.098 *^A^*	0.112 *^A^*
*r*	−0.264	−0.648	0.987	0.095	−0.436	−0.509	−0.126	−0.751

*r*—correlation coefficient. Values with different letters (*^a^*^–*l*^ and *^A–D^*) are significantly different at *p* ≤ 0.01 (Anova, Tukey’s HSD test).

## Data Availability

Data are available by contacting the authors.

## References

[1] Klavins M., Grandovska S., Obuka V., Ievinsh G. (2021). Comparative study of biostimulant properties of industrially and experimentally produced humic substances. Agronomy.

[2] Gümüş İ., Şeker C. (2015). Influence of humic acid applications on modulus of rupture, aggregate stability, electrical conductivity, carbon and nitrogen content of a crusting problem soil. Solid Earth.

[3] Leite J.M., Pitumpe Arachchige P.S., Ciampitti I.A., Hettiarachchi G.M., Maurmann L., Trivelin P.C.O., Prasad P.V.V., Sunoj S.V.J. (2020). Co-addition of humic substances and humic acids with urea enhances foliar nitrogen use efficiency in sugarcane (*Saccharum officinarum* L.). Heliyon.

[4] Stevenson F.J. (1994). Humus Chemistry: Genesis, Composition, Reactions.

[5] Rostami M., Shokouhian A., Mohebodini M. (2022). Effect of humic acid, nitrogen concentrations and application method on the morphological, yield and biochemical characteristics of strawberry ‘Paros’. Int. J. Fruit Sci..

[6] Pena-Mendez M., Havel J., Patocka J. (2005). Humic substances-compounds of still unknown structure: Applications in agriculture, industry, environment, and biomedicine. J. Appl. Biomed..

[7] Abbas T., Ahmad S., Ashraf M., Adnan Shahid M., Yasin M., Mukhtar Balal R., Pervez M.A., Abbas S. (2013). Effect of humic and application at different growth stages of kinnow mandarin (*Citrus reticulata* blanco) on the basis of physio-biochemical and reproductive responses. Acad. J. Biotechnol..

[8] Khan A., Afridi M.Z., Airf M., Ali S., Muhammad I. (2017). A sustainable approach toward maize production: Effectiveness of farmyad manure and urea nitrogen. Ann. Biol. Sci..

[9] Hussain I., Khan A., Akbarm H. (2021). Maize growth in response to beneficial microbes, Humic acid and farmyard manure application. Sarhad J. Agric..

[10] Omonode R.A., Halvorson A.D., Bernard G., Vyn T.J. (2017). Achieving lower nitrogen balance and higher nitrogen recovery efficiency reduces nitrous oxide emissions in North America’s maize cropping systems. Front. Plant Sci..

[11] Ren B., Guo Y., Liu P., Zhao B., Zhang J. (2021). Effects of urea-ammonium nitrate solution on yield, N2O emission, and nitrogen efficiency of summer maize under integration of water and fertilizer. Front. Plant Sci..

[12] Pereira R.V., Filgueiras C.C., Dória J., Peñaflor M.F.G.V., Willett D.S. (2021). The effects of biostimulants on induced plant defense. Front. Agron..

[13] Canellas L.P., Olivares F.L. (2014). Physiological responses to humic substances as plant growth promoter. Chem. Biol. Technol. Agric..

[14] Jindo K., Olivares F.L., Malcher D.J.D.P., Sánchez-Monedero M.A., Kempenaar C., Canellas L.P. (2020). From lab to field: Role of humic substances under open-field and greenhouse conditions as biostimulant and biocontrol agent. Front. Plant Sci..

[15] Ulukan H. (2008). Effect of soil applied humic acid at different sowing times on some yield components of wheat (*Triticum* spp.) hybrids. Int. J. Bot..

[16] Pukalchik M., Kydralieva K., Yakimenko O., Fedoseeva E., Terekhova V. (2019). Outlining the Potential role of humic products in modifying biological properties of the soil—A review. Front. Environ. Sci..

[17] Sharif M., Khattak R.A., Sarir M.S. (2002). Effect of different levels of lignitic coal derived humic acid on growth of maize plants. Commun. Soil Sci. Plant Anal..

[18] Olivares F.L., Aguiar N.O., Rosa R.C.C., Canellas L.P. (2015). Substrate biofortification in combination with foliar sprays of plant growth promoting bacteria and humic substances boosts production of organic tomatoes. Sci. Hortic..

[19] Schoebitz M., López M.D., Serrí H., Martínez O., Zagal E. (2016). Combined application of microbial consortium and humic substances to improve the growth performance of blueberry seedlings. J. Soil Sci. Plant Nutr..

[20] Bharali A., Baruah K.K., Bhattacharyya P., Gorh D. (2017). Integrated nutrient management in wheat grown in a northeast India soil: Impacts on soil organic carbon fractions in relation to grain yield. Soil Tillage Res..

[21] Nardi S., Schiavon M., Francioso O. (2021). Chemical structure and biological activity of humic substances define their role as plant growth promoters. Molecules.

[22] Canellas L.P., Olivares F.L., Aguiar N.O., Jones D.L., Nebbioso A., Mazzei P., Piccolo A. (2015). Humic and fulvic acids as biostimulants in horticulture. Sci. Hortic..

[23] Giovanardi D., Dallai D., Dondini L., Mantovani V., Stefani E. (2016). Elicitation of resistance to bacterial canker of stone fruits by humic and fulvic acids (glucohumates): A cDNA-AFLP-dHPLC approach. Sci. Hortic..

[24] Tahir M.M., Khurshid M., Khan M.Z., Abbasi M.K., Hazmi M.H. (2011). Lignite-derived humic acid effect on growth of wheat plants in different soils. Pedosphere.

[25] Eyheraguibel B., Silvestre J., Morard P. (2008). Effects of humic substances derived from organic waste enhancement on the growth and mineral nutrition of maize. Bioresour. Technol..

[26] Xu S., Zhang L., McLaughlin N.B., Mi J., Chen Q., Liu J. (2015). Effect of synthetic and natural water absorbing soil amendment soil physical properties under potato production in a semi-arid region. Soil Tillage Res..

[27] Shah Z.H., Rehman H.M., Akhtar T., Alsamadany H., Hamooh B.T., Mujtaba T., Daur I., Al Zahrani Y., Alzahrani H.A.S., Ali S. (2018). Humic substances: Determining potential molecular regulatory processes in plants. Front. Plant Sci..

[28] Olivares F.L., Busato J.G., de Paula A.M., da Silva Lima L., Aguiar N.O., Canellas L.P. (2017). Plant growth promoting bacteria and humic substances: Crop promotion and mechanisms of action. Chem. Biol. Technol. Agric..

[29] da Silva M.S.R.A., dos Santos B.d.M.S., da Silva C.S.R.A., da Silva C.S.R.A., Antunes L.F.S., dos Santos R.M., Santos C.H.B., Rigobelo E.C. (2021). Humic substances in combination with plant growth-promoting bacteria as an alternative for sustainable agriculture. Front. Microbiol..

[30] Guo Y., Ma Z., Ren B., Zhao B., Liu P., Zhang J. (2022). Effects of humic acid added to controlled-release fertilizer on summer maize yield, nitrogen use efficiency and greenhouse gas emission. Agriculture.

[31] Susic M., Genc Y., Lyons G. (2016). Replenishing humic acids in agricultural soils. Agronomy.

[32] Wyszkowski M., Brodowska M.S. (2020). Content of trace elements in soil fertilized with potassium and nitrogen. Agriculture.

[33] Brodowska M.S., Wyszkowski M., Bujanowicz-Haraś B. (2022). Mineral fertilization and maize cultivation as factors which determine the content of trace elements in soil. Agronomy.

[34] Wyszkowski M., Brodowska M.S. (2021). Potassium and nitrogen fertilization vs. trace element content of maize (*Zea mays* L.). Agriculture.

[35] Vaccaro S., Ertani A., Nebbioso A., Muscolo A., Quaggiotti S., Piccolo A., Nardi S. (2015). Humic substances stimulate maize nitrogen assimilation and amino acid metabolism at physiological and molecular level. Chem. Biol. Technol. Agric..

[36] Geng J., Yang X., Huo X., Chen J., Lei S., Li H., Lang Y., Liu Q. (2020). Effects of controlled-release urea combined with fulvic acid on soil inorganic nitrogen, leaf senescence and yield of cotton. Sci. Rep..

[37] IUSS Working Group WRB (2015). World Reference Base for Soil Resources 2014; World Soil Resources Report. International Soil Classification System for Naming Soils and Creating Legends for Soil Maps. Update 2015.

[38] Bremner J.M., Norman A.G. (1965). Total nitrogen. Methods of Soil Analysis, Part 2. Chemical and Microbiological Properties (Agronomy 9).

[39] Ostrowska A., Gawliński S., Szczubiałka Z. (1991). Methods for Analysis and Evaluation of Soil and Plant Properties.

[40] Grzesiuk W. (1968). Nephelometric determination of sulphate sulphur in plants. Rocz. Gleboz..

[41] (1998). Soil and Mineral Materials–Sampling and Determination of Particle Size Distribution.

[42] Tibco (2021). Statistica Data Analysis Software System.

[43] Khan S.A., Khan S.U., Qayyum A., Gurmani A.R., Khan A., Khan S.M., Ahmed W., Mehmood A., Amin B.A.Z. (2019). Integration of humic acid with nitrogen wields an auxiliary impact on physiological traits, growth and yield of maize (*Zea mays* L.) varieties. Appl. Ecol. Environ. Res..

[44] Lollato R.P., Figueiredo B.M., Dhillon J.S., Arnall D.B., Raun W.R. (2019). Wheat grain yield and grain-nitrogen relationships as affected by N, P, and K fertilization: A synthesis of long-term experiments. Field Crops Res..

[45] Ichami S.M., Shepherd K.D., Sila A.M., Stoorvogel J.J., Hoffland E. (2019). Fertilizer response and nitrogen use efficiency in African smallholder maize farms. Nutr. Cycl. Agroecosyst..

[46] Ju X.T., Gu B.J. (2014). Status-quo, problem and trend of nitrogen fertilization in China. J. Plant Nutr. Fert..

[47] Zhang W.F., Ma L., Huang G.Q., Wu L., Chen X.P., Zhang F.S. (2013). The development and contribution of nitrogenous fertilizer in China and challenges faced by the country. Sci. Agric. Sin..

[48] Raun W.R., Solie J.B., Johnson G.V., Stone M.L., Mullen R.W., Freeman K.W., Thomason W.E., Lukina E.V. (2002). Improving nitrogen use efficiency in cereal grain production with optical sensing and variable rate application. Agron. J..

[49] Gojon A. (2017). Nitrogen nutrition in plants: Rapid progress and new challenges. J. Exp. Bot..

[50] Kant S., Bi Y., Rothstein S.J. (2011). Understanding plant response to nitrogen limitation for the improvement of crop nitrogen use efficiency. J. Exp. Bot..

[51] Goñi O., Łangowski Ł., Feeney E., Quille P., O’Connell S. (2021). Reducing nitrogen input in barley crops while maintaining yields using an engineered biostimulant derived from *Ascophyllum nodosum* to enhance nitrogen use efficiency. Front. Plant Sci..

[52] Liu X.Y., Yang J.S., Tao J.Y., Yao Y.J. (2022). Integrated application of inorganic fertilizer with fulvic acid for improving soil nutrient supply and nutrient use efficiency of winter wheat in a salt-affected soil. Appl. Soil Ecol..

[53] Azeem K., Naz F., Jalal A., Galindo F.S., Teixeira Filho M.C.M., Khalil F. (2021). Humic acid and nitrogen dose application in corn crop under alkaline soil conditions. Rev. Bras. Eng. Agríc. Ambiental..

[54] Azeem K., Shah S., Ahmad N., Shah S.T., Khan F., Arafat Y., Naz F., Azeem I., Ilyas M. (2015). Physiological indices, biomass and economic yield of maize influenced by humic acid and nitrogen levels. Russ. Agric. Sci..

[55] Baldotto M.A., Melo R.O., Baldotto L.B. (2019). Field corn yield in response to humic acids application in the absence or presence of liming and mineral fertilization. Semina Ciên. Agrár..

[56] Niaz A., Yaseen M., Shakar M., Sultana S., Ehsan M., Nazarat A. (2016). Maize production and nitrogen use efficiency in response to nitrogen application with and without humic acid. J. Anim. Plant Sci..

[57] Suntari R., Retnowati R., Soemarno S., Munir M. (2015). Determination of urea-humic acid dosage of vertisols on the growth and production of rice. AGRIVITA J. Agric. Sci..

[58] Chen X.G., Kou M., Tang Z.H., Zhang A.J., Li H.M. (2017). The use of humic acid urea fertilizer for increasing yield and utilization of nitrogen in sweet potato. Plant Soil Environ..

[59] Zhang S.Q., Yuan L., Li W., Lin Z.A., Li Y.T., Hu S.W., Zhao B.Q. (2019). Effects of urea enhanced with different weathered coal-derived humic acid components on maize yield and fate of fertilizer nitrogen. J. Intergrative Agric..

[60] Pei R.J., Yuan T.Y., Wang J.Z., Hu N., Li Y.N. (2017). Effects of application of humic acid on yield, nitrogen use efficiency of summer maize. Sci. Agric. Sin..

[61] Gao F., Li Z., Du Y., Duan J., Zhang T., Wei Z., Guo L., Gong W., Liu Z., Zhang M. (2022). The combined application of urea and fulvic acid solution improved maize carbon and nitrogen metabolism. Agronomy.

[62] Canellas L.P., Balmori D.M., Médici L.O., Aguiar N.O., Campostrini E., Rosa R.C., Façanha A.R., Olivares F.L. (2013). A combination of humic substances and *Herbaspirillum seropedicae* inoculation enhances the growth of maize (*Zea mays* L.). Plant Soil.

[63] El-Bassioung H.S.M., Bakry B.A., El-Monem Attia A.A., Abd Allah M.M. (2014). Physiological role of humic acid and nicotinamide on improving plant growth, yield, and mineral nutrient of wheat (*Triticum durum*) grown under newly reclaimed sandy soil. Agric. Sci..

[64] Kazemi M. (2013). Effect of foliar application of humic acid and potassium nitrate on cucumber growth. Bull. Environ. Pharmacol. Life Sci..

[65] Mahmoudi M., Samavat S., Mostafavi M., Khalighi A., Cherati A. (2013). The effects of proline and humic acid on quantitative properties of kiwifruit. Int. Res. J. Appl. Basic Sci..

[66] Denre M., Bandopadhyay P.K., Chakravarty A., Pal S., Bhattacharya A. (2014). Effect of foliar application of humic acid, zinc and boron on biochemical changes related to productivity of pungent pepper (*Capsicum annuum* L.). Afr. J. Plant Sci..

[67] Reeza A.A., Ahmed O.H., Majid N.M.N.A., Jalloh M.B. (2009). Reducing ammonia loss from urea by mixing with humic and fulvicacids isolated from coal. Am. J. Environ. Sci..

